# Fontan Failure: Current State of the Art in Mechanical Circulatory Support and Transplantation

**DOI:** 10.3390/children13091215

**Published:** 2026-09-08

**Authors:** Rosaria Barracano, Gabriele De Palma, Claudia Montanaro, Vito Casale, Nunzia Borrelli, Antonella Bruna Cutrì, Micol Rebonato, Ippolita Altobelli, Gianfranco Butera, Giancarlo Scognamiglio, Berardo Sarubbi

**Affiliations:** 1 Adult Congenital Heart Disease Centre, AORN Ospedale dei Colli, Monaldi Hospital, 80131 Naples, Italy; depalma.gabriele97@gmail.com (G.D.P.);; 2 Department of Cardiology, University of Campania Luigi Vanvitelli, 80138 Naples, Italy; 3 Adult Congenital Heart Disease Unit, Bambino Gesù Hospital, Research Institute, 00165 Rome, Italy; 4 National Heart and Lung Institute, Imperial College London, London SW3 6NP, UK; 5 Faculty of Sport Science, University of Naples Partenope, 80138 Naples, Italy

**Keywords:** Fontan failure, advanced heart failure, heart transplantation, mechanical circulatory support

## Abstract

**Highlights:**

**What are the main findings?**

- Despite improved survival, Fontan patients still experience various complications included in the definition of “Fontan failure”;
- A number of them may need to be referred for advanced heart failure therapies.

**What are the implications of the main findings?**

- Advanced heart failure therapies, which include mechanical circulatory support and transplantation, may represent the final stage of palliation for patients with univentricular heart physiology.

**Abstract:**

The Fontan procedure has rewritten the natural history of many children with congenital heart disease and univentricular physiology. However, long-term survival is limited by the development of haemodynamic and multisystem failure, leading to high morbidity and mortality. Absence of a subpulmonary ventricular pump, chronically elevated central venous pressure, and reduced cardiac output, along with lymphatic dysfunction and progressive multiorgan involvement, define the clinical course of many patients with the Fontan procedure, resulting in the heterogeneous clinical entity of Fontan failure. This review provides a comprehensive overview of the pathophysiological mechanisms underlying Fontan failure and a critical analysis of the currently available advanced therapeutic options. Particular attention is paid to the current frontiers of mechanical circulatory support. Although the presence of a total cavopulmonary connection may represent an anatomical limitation, ventricular assist devices have gained attention both as a bridge to transplantation and, in selected cases, as a long-term strategy. Nonetheless, despite the high surgical complexity, heart transplantation currently represents the only definitive option for this patient population. Optimizing clinical outcomes in Fontan failure represents one of the most challenging frontiers in congenital heart disease. Achieving significant advances will require accurate risk stratification, timely transition to advanced therapies, and coordinated multidisciplinary collaboration.

## 1. Introduction

The Fontan procedure has allowed the treatment of patients with single-ventricle heart physiology, offering a palliative option to those who would otherwise face a dismal prognosis. Over the years, advancements in surgical techniques, perioperative care, and long-term management have significantly improved survival rates, currently reported to be up to 80–90% at 30 years of age [1]. However, as patients with a Fontan circulation grow, a new set of challenges emerge. Many of these individuals experience various complications such as heart failure (HF), arrhythmias, and multiorgan dysfunction, which are often refractory to medical therapy [1,2,3]. In this context, mechanical circulatory support (MCS) and heart transplantation (HT) have become essential tools and treatment options for patients with failing Fontan circulation. Unfortunately, the unique anatomical and physiological characteristics make both MCS and HT technically demanding and associated with higher morbidity and mortality compared to patients with biventricular circulation [4,5,6]. In some cases, Fontan patients may require combined heart and liver transplantation, which poses additional challenges due to the complexity of the procedure and the further scarcity of donor organs.

This review aims to provide an overview of the current state of the art in MCS and transplantation for Fontan patients, highlighting the challenges, controversies, and future directions in this rapidly evolving field. The discussion will focus on the most recent advances in MCS, including indication and configuration of available ventricular assist devices (VADs). Additionally, we will examine current candidate selection for advanced hear failure therapies (AHFTs) and relative multidisciplinary assessment. Through this comprehensive review, we aim to provide clinicians and researchers with a deeper understanding of the complexities involved in managing patients with Fontan failure.

## 2. Mechanisms of Fontan Failure

Fontan failure is a heterogeneous clinical syndrome, including both cardiac and extracardiac complications, arising from a combination of circulatory and end-organ dysfunction. Multiple mechanisms are involved, including ventricular systolic and diastolic dysfunction, atrioventricular valve regurgitation (AVVR), increased pulmonary vascular resistance (PVR), recurrent arrhythmias, Fontan pathway obstruction, lymphatic abnormalities, and progressive end-organ dysfunction [1,2,4,5].

Fontan circulation is characterized by the lack of a pumping subpulmonic ventricle: in this context, forward flow through pulmonary circulation depends entirely on a non-pulsatile pressure gradient, requiring central venous pressure (CVP) to exceed pulmonary arterial and left atrial pressures. This haemodynamic configuration restricts pulmonary blood delivery, reduces preload of the systemic ventricle, and results in a persistently low cardiac output (CO) state with elevated CVP. Gewillig et al. conceptualized the Fontan pathway as a haemodynamic bottleneck limiting forward flow. In systems characterized by serial resistances, overall flow is constrained by the segment with the highest resistance. As a result, elevations in PVR, Fontan pathway resistance or end-diastolic pressure (EDP) may lead to clinically significant venous congestion and reduced CO [2].

Cyanosis is common among Fontan patients, with causes including the presence of conduit fenestration, pulmonary arteriovenous malformations (PAVMs), and veno-venous collaterals (VVCs). Liver-derived factors, together with non-pulsatile pulmonary blood flow and hypoxia, have been associated with PAVM development. VVCs are frequent findings as well and, even if their genesis has not been properly understood, de novo angiogenesis or, more plausibly, reopening of preexisting venous channels due to elevated CVP are postulated causes. Aortopulmonary collaterals (APCs) may be encountered as well, leading to systemic ventricular overload and increased CVP [1,7].

In the Fontan circulation, chronic preload deprivation may initiate a maladaptive cycle of ventricular remodelling, reduced compliance, and progressively elevated filling pressures, therefore exacerbating the preload mismatch and functional impairment. Consequently, elevated EDP further limits transpulmonary flow, perpetuating a vicious cycle of declining CO and worsening venous congestion [2].

Furthermore, significant AVVR may contribute to Fontan failure by increasing EDP and reducing effective stroke volume [2]. Arrhythmias represent another key determinant and should be regarded not only as a consequence of the failing Fontan circulation, but also as a potential trigger of clinical worsening [1,3]. Fontan pathway stenosis may also be considered an underlying mechanism of Fontan failure by causing obstruction, increasing CVP and reducing ventricular preload [1,2,5].

Finally, extracardiac complications result from the combination of chronically elevated CVP and reduced CO, which leads to an impairment of organ perfusion and persistent systemic venous congestion. At the hepatic level, these mechanisms promote sinusoidal congestion, chronic hypoperfusion, and progressive fibrosis, leading to FALD. Chronic cyanosis contributes as well to FALD, since decreased arterial oxygenation exacerbates hepatic sinusoidal congestion and hypoxia, accelerating microvascular remodelling, hepatic hypoperfusion, and progressive fibrosis [8]. Concurrently, chronically reduced renal perfusion (from low CO), associated with increased CVP and chronic hypoxia, contributes to the development of renal dysfunction and reduced glomerular filtration rate by impairing the glomerular filtration barrier [3,4]. This predisposes Fontan patients to proteinuria and long-term chronic kidney disease [9].

Protein-losing enteropathy (PLE) represents one of the most severe manifestations of Fontan failure and is an expression of the complex interaction between haemodynamic impairment and lymphatic dysfunction. Elevated CVP impairs intestinal lymphatic drainage, while low CO promotes mesenteric vasoconstriction, hypoperfusion, and intestinal mucosa inflammation. These factors contribute to impaired enterocyte barrier integrity, leading to leakage of serum proteins into the intestinal lumen, resulting in diarrhea, edema, and immune abnormalities [10]. Plastic bronchitis (PB) is a rare, but potentially fatal, complication of Fontan failure with a poorly understood pathophysiology. Current evidence suggests that it is caused by lymphatic abnormalities in the context of venous congestion and low CO, with a possible contribution from an abnormal inflammatory response [1,3,4].

## 3. Referral and Evaluation for Advanced Heart Failure Therapies

Indications and timing for AHFT for failing Fontan are still not clearly established. Early referral is crucial, since the onset of Fontan failure (especially extracardiac morbidities) dramatically impacts death and HT-free survival, and its complications can compromise candidacy, waitlist and post-HT survival [5]. Indeed, late referral for AHFT is encountered in up to 40% of Fontan patients, while a significant proportion die in the waitlist or are declined due to advanced disease [5,11,12].

The ACTION committee has developed criteria for AHFT referral (Table 1) to avoid late referral and allow for a comprehensive multidisciplinary assessment in anticipation of AHFT [3]. Identifying the progression and refractoriness of Fontan complications (i.e., exercise performance decline, fluid overload, PLE, and cyanosis) represents an essential part of AHFT referral, as highlighted by the ACTION criteria. Furthermore, failure of alternative available therapies and timely interception of the declining trajectory of failing Fontan patients are key factors for appropriate timing for AHFT.

A comprehensive multidisciplinary assessment should be performed to assess the presence and the severity of all Fontan failure “components”. Moreover, potential reversible causes of Fontan failure and suitability for alternative or bridging therapies, including interventional treatments, need to be addressed, as well as all the factors that impact patient clinical status and management before and/or after AHFT (i.e., restrictive lung disease, nutritional status, risk of infections). Optimizing patient clinical status is crucial to improve AHFT outcomes. A summary list of items included in the multidisciplinary assessment for AHFT is illustrated in Table 2 [3,4,5,6,13,14].

Inotropes, both with intermittent or continuous infusion (respectively with levosimendan and milrinone as the most adopted inotropes), has been widely adopted to bridge patients with Fontan failure to HT [15], with up to 70% of them receiving inotropes at the time of HT. Milrinone and levosimendan can improve Fontan haemodynamic condition, enhancing organ perfusion and reducing venous congestion through their inotropic and lusitropic effect and their vasodilatory action on pulmonary and systemic vasculature [15,16]. However, evidence of improving or stabilizing Fontan-related complications is still poor. Since haemodynamic benefit may be limited, appropriate patient selection is crucial, especially in those without systemic ventricle dysfunction [2].

Preoperative cross-sectional imaging is crucial in surgical planning for both HT and long-term VAD, given the anatomical and technical complexity [4,5,17]. Virtual simulations can provide additional help, especially for VAD implantation [4].

A detailed assessment of the haemodynamic phenotype of Fontan failure is essential to evaluate the underlying mechanisms and its haemodynamic severity, which are both crucial for AHFT referral, as well as for eligibility for HT and long-term VAD [3,4,5].

The estimation of PVR is crucial for HT assessment, since even mildly elevated PVR increases the risk of postoperative graft failure [5,13]. Moreover, it is relevant for HT candidacy and VAD configuration [4,17]. Nonetheless, calculating PVR at cardiac catheterization (CC) may be challenging due to multiple factors, such as low CO, the presence of fenestration and unequal or multiple pulmonary blood flow sources. Combined cardiac magnetic resonance (CMR) may be helpful, allowing simultaneous assessment of anatomy and function and quantification of flows, with a better accuracy than the standard Fick Method [1]. Moreover, rapid volume loading and exercise CC are useful tools to unmask occult diastolic dysfunction and provide additional dynamic information on pulmonary vasculature [3,4,18].

Assessment of collateral blood vessel burden is another key step before AHFT. APCs significantly increase the risk of surgically bleeding and can lead to persistent overload of the left ventricle post-HT. Aggressive catheter-based embolization of APCs pre-transplant has been shown to improve outcomes and is generally performed [6,19]. In VAD recipients, higher pump flows may allow compensation for pulmonary shunting while maintaining adequate systemic output. However, patients with larger APC burden may need prior embolization, since pulmonary artery pressure may increase excessively, and even elevated flows may not be enough to compensate for this [4]. Compared to APCs, the association between VVCs and intraoperative bleeding is not so fully established, yet they can cause persistent desaturation, which may lead to unnecessary vasoactive therapies and prolonged ventilation [5,6,12]. Embolization could be indicated according to the extent of VVCs and the risk of increasing Fontan pressures. A tailored approach including close patient monitoring, serial assessment of VVC status and percutaneous closure could be useful [6,12]. Management of Fontan fenestration is still a debated issue in VAD recipients. Despite the risk of systemic thromboembolism and desaturation, leaving or creating a fenestration may be reasonable, since it can represent an important preload source, particularly in the delicate postoperative period when positive pressure ventilation and labile PVR can further reduce preload before circulatory adaptation to MCS, especially in patients with concerns of inadequate VAD filling. Previous reports showed no increased risk of stroke, and arterial saturation tended to rise with improved CO and decreased CVP, while the fenestration could be percutaneously closed postoperatively if indicated [4,14,20,21].

Multiorgan dysfunction needs to be extensively investigated with multidisciplinary involvement, since it not only represents an important indication for AHFT referral, but also significantly impacts pre-, intra- and postoperative management and outcomes [3,4,5,22]. Deciding whether a patient may benefit from heart-only or multiorgan transplantation is again challenging and fraught with little data to support or refute any given approach [17]. In particular, a debated topic is the indication for CHLT. Despite some reports even describing a partial reversal [23], the fate of hepatic abnormalities and the potential of liver recovery after restoration of normal haemodynamic function with HT are largely unknown, especially in patients with more advanced FALD [24]. CHLT has shown positive results [12,22,25], with an overall survival at 1 and 5 years respectively of up to 89% and 84% in selected patients in the current era [22]. It may be associated with better long-term outcomes compared with isolated HT, especially in those with more advanced FALD [22]. Moreover, CHTL shows a favourable immunomodulatory response with a lower rejection rate. Nonetheless, CHLT implies increased technical complexity, with higher postoperative complications and mortality, while superiority to isolated HT in the overall FALD population is still unclear [22,25]. Therefore, appropriate patient selection is crucial, with definite criteria still lacking.

In patients without heart disease, the MELD score is used to determine the severity of liver disease and liver transplant waiting list status. While the modified MELD-XI score correlates with perioperative morbidity and post-transplant survival, it does not provide a validated threshold for the failing Fontan population [25,26], where multisystemic complications may affect MELD components, such as albumin and creatinine, irrespective of the severity of FALD.

Liver biopsy may be a useful tool for diagnosis and staging of liver fibrosis, with histological evidence of more advanced disease used as a criterion for CHLT referral in some centres [22,25]. However, several limitations are present, such as the patchy nature of FALD and a non-negligible risk of complications, whereas its prognostic value for candidate selection for CHLT is still poorly established [26,27].

The presence of overt complications of portal hypertension has also been used to identify patients with more advanced FALD who may benefit from CHLT [22]. In particular, a recent multicentre study showed higher survival rates with CHLT compared to isolated HT in patients with at least two of the following: liver cirrhosis, splenomegaly, varices and ≥2 paracentesis [22]. Still, failing Fontan patients may present confounding factors contributing to these complications, such as ascites, that may not entirely reflect the severity of FALD itself.

Hepatocellular carcinoma represents probably the most severe complication of FALD, with a dramatic impact on survival [27]. Concomitant presence of hepatocellular carcinoma suitable for liver transplantation represents a strong indication for CHLT [25].

Fontan patients often display high levels of human leukocyte antigen (HLA) antibodies due to homograft material and blood product exposure, which may increase the risk of graft dysfunction and rejection and prolong waitlist times to avoid crossing donor-specific HLA antibodies. Early referral and desensitization protocols are therefore important strategies [5,6].

## 4. Mechanical Circulatory Support

Mechanical circulatory support is progressively emerging as a viable strategy in patients with Fontan failure requiring AHFT, both in the acute and chronic setting.

**Figure 1:**
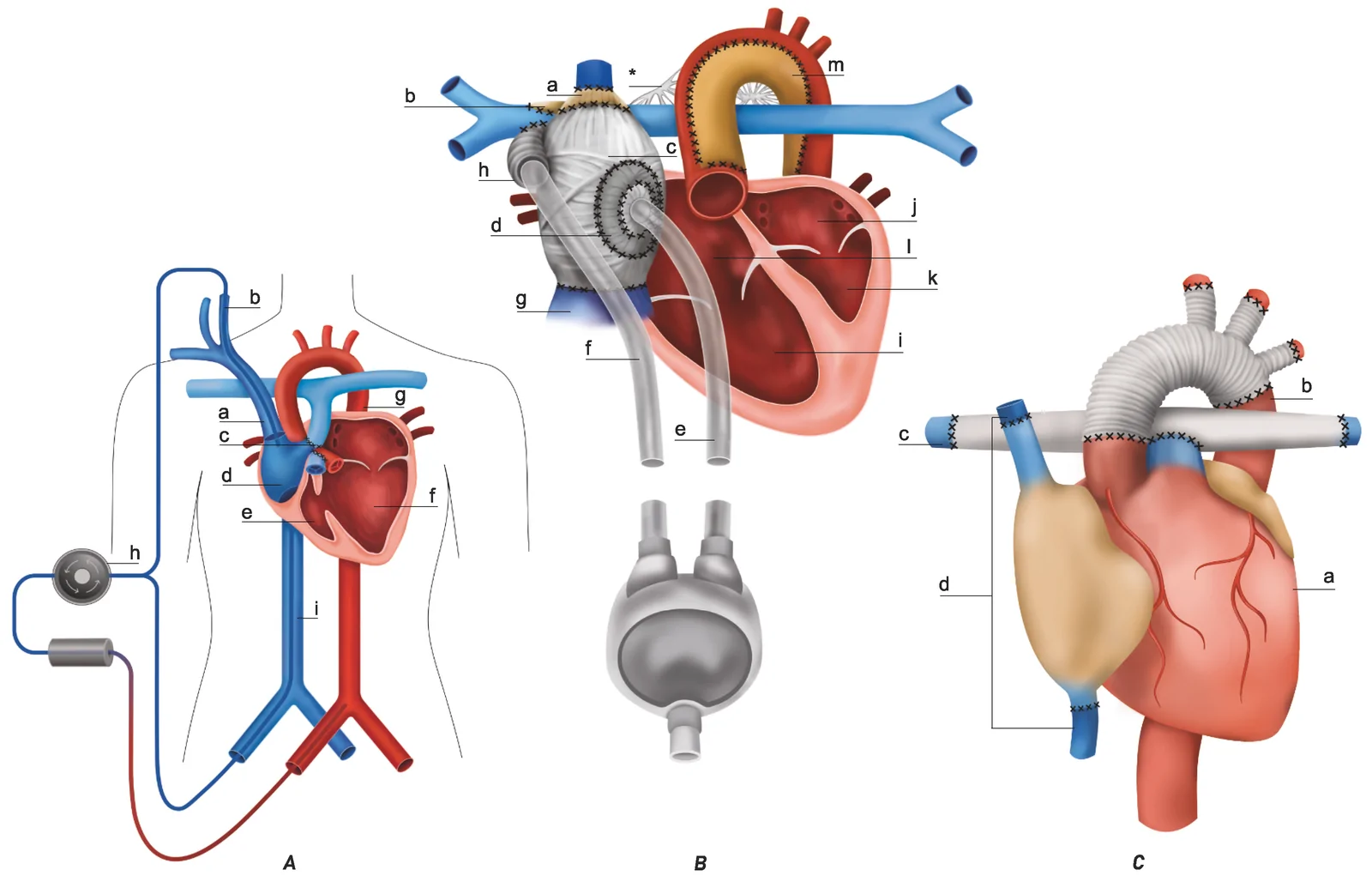
Graphic illustration of the surgical complexity of mechanical circulatory support and heart transplantation in the Fontan population. (**A**) = Tricuspid atresia and atriopulmonary Fontan with veno-arterial extracorporeal membrane oxygenation with a double venous cannula (h) in the superior (a) and inferior vena cava (i). b = Right internal jugular vein; c = pulmonary artery—right atrium anastomosis; d = tricuspid atresia; e = right ventricle; f = left ventricle; g = aorta. (**B**) = Hypoplastic left heart syndrome and stage III Norwood operation with extracardiac Fontan with a subpulmonary ventricular assist device requiring the construction of an “artificial right atrium” with a Dacron graft (c) anastomosed directly with the inferior vena cava (g) and connected to the inflow cannula (e) via an oval Dacron patch (d) and to the superior vena cava via a bovine pericardial patch (a). The Glenn anastomosis is taken down, with the pulmonary branch repaired using a bovine pericardial patch (b); the Fontan conduit is taken down, leaving the distal part (h) connected to the outflow cannula (f). i = Right ventricle; j = left atrium; k = left ventricle; l = right atrium; m = dilated neo-aorta with multiple adherences with the pulmonary branches (asterisk). Adapted from Moosmann et al. [40]. (**C**) = Heart transplant in hypoplastic left heart syndrome and a stage III Norwood operation with extracardiac Fontan requiring en block removal of the ascending aorta, aortic arch and central pulmonary arteries. The ascending aorta and aortic arch have been reconstructed with a vascular Dacron graft (b) sutured to the descending aorta; hilum-to-hilum pulmonary artery reconstruction has been performed with a homograft (c); systemic venous pathways have been reconstructed with donor tissue connected to the recipient superior and inferior vena cava (d). a = Donor heart. Adapted from Konstantinov et al. [17].

Veno-arterial extracorporeal membrane oxygenation (VA-ECMO) is the most adopted device in the acute setting (Figure 1), providing full haemodynamic and respiratory support with a relatively easy and rapid insertion, although peripheral access can be more challenging than for the non-CHD population [20,28]. However, VA-ECMO has an extremely high rate of major complications and mortality, especially in Fontan patients, where the reported discharge rate is 35% [29] and does not allow extubation and mobilization [20]. Therefore, its use should be limited when alternative supports are not feasible and for the shortest time possible [30]. VA-ECMO does not provide an effective unloading of the systemic ventricle, which is particularly relevant in Fontan patients, who often need higher flows to maintain adequate output due to low native CO, thus increasing afterload. Furthermore, APCs can lead to consequent blood recirculation and increased EDP [28,30]. To partially overcome these issues, an unloading strategy may be useful. In addition, in patients undergoing concomitant cardiac surgery, central implantation of the arterial cannula in the ascending aorta can be adopted [28,30]. Moreover, the lack of a subpulmonic ventricle may lead to inadequate venous drainage in the setting of peripheral cannulation, with the superior caval flow directly draining into the pulmonary artery. In these cases, the use of a double venous cannula to successfully decompress the Fontan circulation may be required [28,30].

Impella devices are progressively emerging in the CHD setting, with promising results found from a small number of reports in Fontan patients [31,32]. The Impella devices are micro-axial continuous flow pumps positioned across the aortic valve in the ventricular cavity. Currently available devices are the percutaneously inserted Impella CP (14 Fr, flow of up to 3.7 L/min), and the Impella 5.5 (21 Fr, flow of up to 5.5 L/min), which requires a minimally invasive surgical approach (generally a trans-axillary implantation), but allows early mobilization and patient rehabilitation. While systemic ventricle thrombus and mechanical aortic prothesis are absolute contraindications, the intracardiac anatomy of Fontan patients generally does not limit device implantation. Impella devices actively drain blood from the systemic ventricle to the aorta, guaranteeing anterograde flow to support organs and coronary perfusion, while effectively lowering ventricular EDP and ventricular wall tension, thus reducing myocardial workload, decreasing myocardial oxygen demand and improving myocardial perfusion. On the other hand, Impella devices provide only systemic ventricle support and are strongly dependent on the systemic ventricle preload. Impella devices may be a reasonable option for de-escalation or as an alternative to VA-ECMO in patients with isolated systolic dysfunction, obtaining an effective unloading of the systemic ventricle and allowing early extubation and eventually mobilization [32]. Moreover, they may constitute a “haemodynamic test” for suitability to long-term systemic ventricle VAD implantation [33], sharing a similar haemodynamic profile with left ventricular VAD, especially the Impella 5.5 [34].

The natural history of Fontan palliation, along with the shortage of HT donors and the long waitlist time, represent an attractive setting for the application of long-term MCS. Indeed, MCS may hamper the progression of Fontan failure and related complications or even reverse them, similarly to HT. Therefore, long-term MCS could be a relevant strategy in Fontan failure for bridge-to-transplant (BTT) or bridge-to-candidacy (BTC) strategies, allowing patients to undergo transplantation in better conditions, possibly reducing waitlist mortality, improving post-HT outcomes, and in some cases enabling listing in previously excluded patients [4,17,20,35]. On the other hand, the use of MCS as a destination therapy or bridge-to-recovery strategy has been limited to isolated cases [35,36,37]. The most common implanted devices and their relative specific features are described in Table 3 [14,20,21].

Larger multicentric observational studies have recently shown encouraging results of MCS in both pediatric and adult Fontan populations, with most patients undergoing transplant or being alive with the device implanted (up to 70 and 27% respectively) at 12 months, despite a non-negligible percentage of mortality (18–22%) and complications [35,36,37].

Nonetheless, a large proportion of patients seem to receive MCS at a critical stage (up to 30% in INTERMACS profile 1 and 29% on VA-ECMO) [35,36,37], though with a negative prognostic impact. This points out a late referral issue for long-term MCS in this population, limiting the potential benefit of this strategy [4].

However, the advantages of early referral need to be weighed against the technical and anatomical complexity of VAD placement and the inherent risk of short- and long-term complications in this population.

Beyond the inherent difficulties of VAD implantation in small-sized patients, limiting the device choice and increasing the rate of complications [20,38], surgical complexity is further enhanced by the peculiar anatomy of Fontan patients, previous multiple sternotomies, haemostatic disorders and multisystemic dysfunction often observed in this cohort, especially at the time of VAD referral [4,20].

The unique haemodynamic profile of Fontan failure and its extreme variability further complicate the selection of appropriate candidates for long-term MCS and tailoring of optimal VAD configuration, which includes systemic ventricle VAD (SVAD), subpulmonary VAD (SpVAD) or biventricular VAD (BiVAD) [4].

For SVAD, positioning of the inflow cannula may be challenging. The smaller ventricular dimensions and the variable ventricular morphology and orientation, often with increased trabeculation and redundancy of the subvalvular AV apparatus and absence of a “true apex”, can lead to incorrect placement of the inflow cannula with distortion of the VAD system, increasing the risk of inadequate drainage, suction events and pump thrombosis.

Several approaches have been proposed, such as using the systemic ventricle apex, the diaphragmatic surface, and, less commonly, atrial cannulation, according to the specific anatomic features. Additional dislocation or resection of the AV valvular or subvalvular apparatus and/or trabeculae have been variably adopted [4,20,21].

In intracorporeal VAD, different sites and strategies for allocation of the pump in the chest have been proposed, such as diaphragm and costal cartilage modifications [4,20]. In case of relevant regurgitation of the systemic valve, its repair or oversewing is necessary, usually with a low threshold of intervention [21].

Implantation of an SpVAD or BiVAD implies a demolitive approach, necessitating the breakdown of the cavopulmonary connections, partitioning the systemic and pulmonary circulations and creating a “collecting chamber” suitable for positioning the inflow cannula [20,39,40,41,42] (Figure 1). Dedicated inflow cannulae, like the Berlin Heart EXCOR Venous Cannula, may reduce technical complexity, allowing venous systemic cannulation without the necessity of creating a collecting chamber, with encouraging results in the first reports [43,44].

The optimal haemodynamic profile for SVAD adoption is severe systolic dysfunction of the systemic ventricle or moderate systolic dysfunction associated with moderate to severe AVVR (not suitable for valve interventions) in the presence of elevated EDP [4,14,21].

In this setting, unloading the single ventricle with SVAD may be indicated as BTT or BTC in patients with progressive HF symptoms (i.e., HF hospitalization, declining functional class or exercise performance, severe growth restriction) unresponsive to medical therapy, or progressive organ dysfunction related to systolic failure [4,20].

Application of SVAD in isolated diastolic dysfunction is still a matter of debate, with significant “preload” concerns and implantation challenges in small systemic ventricles, possibly necessitating BiVAD [4,14].

Conversely, in patients with Fontan failure and preserved systemic ventricle function or mixed phenotypes, SVAD may not be helpful, since the augmentation of CO cannot be matched in the failing subpulmonary circulation, despite the reduction in EDP, leading to subpulmonary overloading and lack of adequate preload of the SVAD. In this population, implantation of an SpVAD or, alternatively, a BiVAD, especially in those with mixed phenotypes or elevated PVR, may be the only solution [4,14,21]. However, the high surgical complexity and the significant risk of complications only make this approach reasonable for more advanced patients with progressive and refractory Fontan failure, with current evidence limited to fewer cases compared to SVAD [35,36,37,39,40,41,42,43,44].

**Figure 2:**
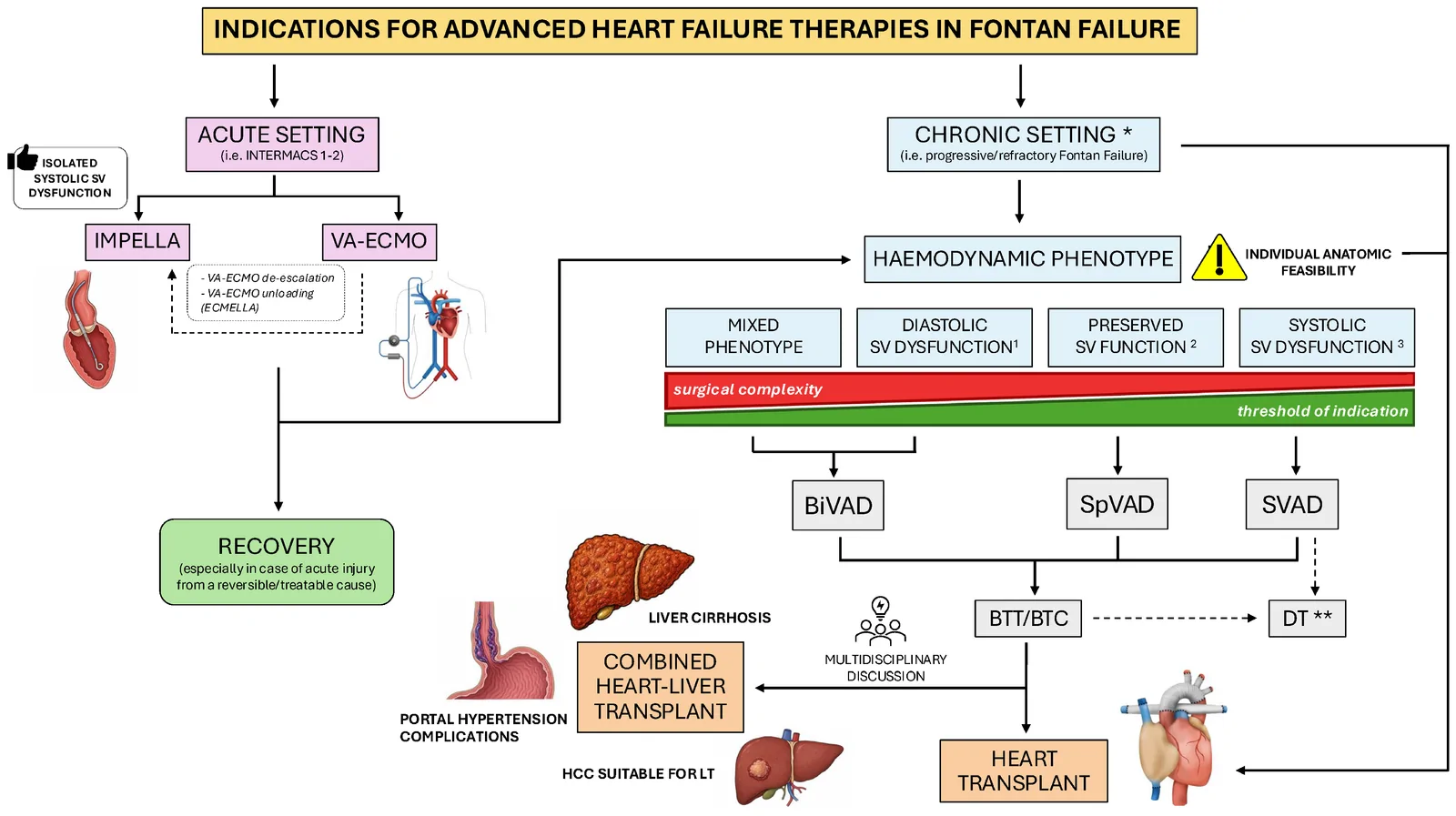
Proposed algorithm for advanced heart failure therapies for failing Fontan circulation (with a focus on mechanical circulatory support in acute and chronic settings). List of abbreviations: BiVAD = biventricular assist device; BTC = bridge to candidacy; BTT = bridge to transplant; DT = destination therapy; HCC: hepatocellular carcinoma; LT = liver transplant; SV = single ventricle; SVAD = systemic ventricular assist device; SpVAD = subpulmonary assist device; VA-ECMO = veno-arterial extracorporeal membrane oxygenation; * = For the definition of progressive and refractory Fontan failure see the ACTION criteria (Table 1) and the relative paragraph. ** = The use of ventricular assist devices as destination therapy has been limited to isolated cases [31,32]. ^1^ Defined as elevated end-diastolic pressure without impaired systolic function. ^2^ This definition applies to patients with progressive and refractory Fontan failure who do not fulfil criteria for systolic/diastolic dysfunction. ^3^ Defined as severe systolic dysfunction of the systemic ventricle or moderate systolic dysfunction associated with moderate to severe atrioventricular valve regurgitation (not suitable for valve interventions) in the presence of elevated end-diastolic pressure.

In this setting, cavopulmonary assist devices implanted in the Fontan circuit to support the subpulmonary circulation as a “neo-right ventricle”, suitable for the anatomy of the Fontan pathway and the haemodynamic of low pressure subpulmonary circulation, is a fascinating strategy, but is still at a preclinical level [14]. A proposed algorithm is illustrated in Figure 2.

## 5. Heart Transplantation

With increasing survival into adulthood, HT has emerged as the definitive “fourth stage” of palliation for many patients with univentricular heart physiology [5]. Owing to the lack of evidence-based medical therapy, transplant is often the only effective treatment for Fontan circulatory failure and related complications, irrespective of their mechanism. PLE and PB generally resolve after HT. Sporadic recurrences have been described for PLE, typically related to transient factors and followed by a complete resolution with their correction [45,46]. Survival rates after HT have improved significantly, with one-year survival now exceeding 90% in recent multicentre studies, and 5-year survival ranging from 70% to 80%, with the greatest risk still in the postoperative period [12,17,19,47].

However, HT is a complex procedure, with a high risk of perioperative complications due to Fontan-specific risk factors, in particular multiorgan dysfunction, complex anatomies requiring technically demanding surgery, predisposition to intense vasoplegia, abnormal pulmonary vascular response, longer cardiopulmonary bypass (CBP) and graft ischemic times, and high bleeding rates, all significantly impacting outcomes.

Crucial contributing factors to surgical complexity are multiple prior sternotomies, with adherences and distorted anatomies, and the frequent requirement of complex vascular reconstructions, entailing elaborate surgical planning. A previous Norwood procedure has a considerable impact, since it generally requires complex aortic arch reconstructions and extensive resections, due to dense and diffuse adherences involving the great vessels [17] (Figure 1). Anomalies of systemic venous returns further increase surgical complexity, especially in patients with inferior vena cava interruption, with several techniques used for systemic pathway reconstruction, adopting both donor and prosthetic material [17,48]. Pulmonary artery reconstructions of various degrees are generally performed, with additional challenges derived from the presence of branch stents, previously modified Blalock–Taussig shunts and tenuous adherences [17,48]. Anomalies of the atrial morphology can pose further difficulties in atrial preparation for anastomosis [48]. Time from operating room entry to readiness for CBP routinely exceeds four hours [17]. Therefore, dissection and vascular reconstructions should be performed before donor heart arrival to minimize graft ischemic time, but meticulous donor–recipient coordination is essential [6,12,13,17].

Perioperative bleeding critically impacts peri-transplant mortality [12,49]. Fontan patients exhibit a higher bleeding risk in relation to pre-existing coagulopathy, blood collaterals, complex surgical procedures and a long CBP [6,12,49].

Weaning from CBP and maintaining haemodynamic stability may be challenging, often requiring high-dose inotropic and chronotropic agents as well as vasopressors [19,50,51]. Vasoplegia can be a relevant issue, promoted by non-modifiable factors such as intrinsic alterations of the vascular bed (related to endothelial dysfunction, abnormal vascular response, and liver dysfunction), and additional modifiable factors, such as long CBP times [19,22,51,52]. Vasoplegia can be particularly sustained and less responsive to vasopressors, and it is associated with negative outcomes [12,19,51].

Postoperative course can be complicated by abnormal pulmonary vascular response, possibly related to occult pulmonary vascular disease manifesting after HT, reduced compliance to sudden changes in pulmonary flow, and precipitating perioperative factors affecting PVR, such as mechanical ventilation, hypercapnia, hypoxemia, and acidosis [50,51,52,53,54]. Nitric oxide is generally the pulmonary vasodilator of choice [13,49,50], owing to its local action without significant impact on the ventilation/perfusion ratio [55], and it is usually started after separation form CBP [13,50]. In case of necessity of inotropic support, milrinone could be the inotrope of choice due to its vasodilatatory effect of pulmonary vasculature [56]. However, caution must be paid in case of significant vasoplegia because of its concomitant vasodilatatory effect on systemic vasculature.

Other important postoperative issues are volume status and renal function, requiring intensive monitoring and rapid optimization [6,50] with the prompt adoption of continuous renal replacement therapy to hamper the haemodynamic impact of acute kidney injury [6].

The perioperative management of a CHLT is even more complex due to the pre-existing liver dysfunction and coagulopathy, the increased surgical complexity of this procedure and the higher rates of bleeding and vasoplegia [6,50,52,57]. Maintenance of an adequate transpulmonary gradient is crucial until initiation of CBP and after reperfusion of the hepatic graft, which, in addition, may have a detrimental impact on PVR. An adequate preload is a key determinant of pulmonary blood flow and CO; therefore it should be strictly monitored and promptly corrected, especially in case of sudden variations, such as bleeding. PVR may be minimized by aggressive management of hypercapnia, hypoxemia, and acidosis, and utilization of pulmonary vasodilators when needed, in particular nitric oxide [50]. Vasopressors should be wisely chosen when indicated, with vasopressin preferred due to its limited effect on PVR [19,50]. Atrial arrhythmias are often poorly haemodynamically tolerated and require prompt correction, usually with electrical cardioversion or pacing [50]. Avoiding hepatic venous congestion and optimal reperfusion of the hepatic graft and aiming to achieve an adequate mean arterial pressure before and after hepatic artery anastomosis are crucial steps [6,50,57]. Close haemodynamic monitoring and support, with prompt adoption of MCS in case of unresponsive deterioration, are paramount, considering the detrimental vicious circle that could be established between the interaction of the liver and cardiac graft failure [57].

Regarding long-term complications, rejection rates are comparable to other CHDs, with higher risk in sensitized patients [13]. Infection rates are higher, especially in patients with PLE or lymphopenia (up to 54% at one-year post-transplant) [13,58], and this represents a leading cause of death and morbidity [59]. In addition, some reports have described an increased risk of malignancy [35], though this is not universally reported.

## 6. Conclusions

Despite their increased survival, Fontan patients still experience several complications, often requiring AHFT. MCS may represent a valuable option in the acute setting or for selected patients for BTT or BTC. However, MCS is technically challenging in this population, with a significant risk of complications, while appropriate timing and indication are still debated. In this scenario, HT may represent the only definitive option for these patients. Nonetheless, late referral is common and both early and late post-transplant complications may occur. Continued innovation in perioperative strategies, care standardization, accurate risk stratification and patient selection, timely transition to advanced therapies, and coordinated multidisciplinary collaboration, including among ACHD cardiologists, HF cardiologists, HT cardiac surgeons, intensivists, anesthetists, and liver and infective disease specialists, are essential to further the care of and improve outcomes for this expanding yet nevertheless complex population.

## Figures and Tables

**Table 1:** ACTION criteria for referral for advanced heart failure therapies by type of clinical Fontan dysfunction.

**Cardiac systemic ventricular dysfunction**
Severe systolic dysfunction ^1^ by TTE or CMR or severe ventricular dilation ^2^ by CMR; Moderately depressed systolic function (by qualitative assessment on imaging) when accompanied by AVVR ≥ moderate; Significant growth derangements including cachexia and linear growth failure ^3^; Progressive decline in exercise performance (by patient report or on sequential CPET or 6MWT); Significant electrophysiologic abnormalities, including recurrent arrhythmias despite therapy, implantation of a PM, or aborted SCD.
**Fontan pathway dysfunction**
Symptomatic, chronic fluid overload persisting despite new or increasing diuretic therapy ^4^; Chronic pleural effusions or ascites that are chylous or non-chylous, refractory to therapy, and occur outside the initial Fontan postoperative period; Major haemodynamic disturbances resulting in symptoms despite therapy, including low systemic cardiac index, diastolic dysfunction, elevated Fontan pressure, or symptomatic cyanosis ^5^.
**Lymphatic dysfunction**
PLE that has failed medical therapy ^5^; Plastic bronchitis requiring chronic therapy or inpatient hospital admission.
**Extracardiac dysfunction**
FALD with impaired synthetic function/abnormal liver function testing or those undergoing evaluation for liver transplantation or hepatocellular carcinoma ^6^; CKD ≥ stage III (eGFR < 60 mL/min/1.73 m^2^); Haemoptysis requiring evaluation that is unrelated to an infection and persists after standard intervention.

^1^ *Severe systolic function defined as EF < 35% for morphologically LV and <30% for morphologically RV.* ^2^ *Severe systemic ventricular dilatation defined as EVDi > 156 mL/BSA.* ^3^ *Examples of significant growth derangements are deviation in the height-for-age curve downward across two major height percentile curves or prepubertal height velocity < 4 cm/y.* ^4^ *Examples of major haemodynamic alterations are CI < 2 L/min/m^2^, EDP > 15 mmHg and Fontan pressure ≥ 20 mmHg.* ^5^ *Examples of refractory PLE are at least one related hospital admission in a 12 mo period, requirement of repeated albumin infusions or steroid side effects from ongoing budesonide therapy with failure of other therapies.* ^6^ *The EASL-ERN position paper states that heart transplant should not be considered in advanced FALD per se, but rather in case of FALD progression that might indicate haemodynamic deterioration. List of abbreviations: AVVR = atrioventricular valve regurgitation; CI = cardiac index; CKD = chronic kidney disease; CMR = cardiac magnetic resonance; CPET = cardiopulmonary exercise testing; EDP = end-diastolic pressure; EDVi = end-diastolic volume indexed; EF = ejection fraction; FALD = Fontan-associated liver disease; LV = left ventricle; PLE = protein-losing enteropathy; PM = pacemaker; TTE = transthoracic echocardiography; RV = right ventricle; 6MWT = 6 min walking test. Adapted from Lubert et al. [3]*.

**Table 2:** Multidisciplinary evaluation for advanced heart failure therapy referral.

Items	Diagnostic Tests	Specific Considerations
**Surgical planning**	Cross-sectional imaging (CT/CMR) Virtual 3D simulation (for long-term VAD)	Comprehensive evaluation of peripheral accesses, cardiac anatomy (i.e., situs inversus, dextrocardia, great vessel status and orientation, systemic or pulmonary venous return abnormalities, coronary anatomy), extracardiac thoracic anatomy and adherences
**Blood vessel collaterals**	Cross-section imaging (CT, CMR) Cardiac catheterization	CMR allows precise quantification of overall collateral flow
**Haemodynamic evaluation**	TTE *(for systemic ventricle and AV function, systemic outflow tract and Fontan pathway obstruction)* TEE *(for characterization of significant AV regurgitation)*	Echocardiography alone provides a limited evaluation of systemic ventricle systolic function and volumes due to heterogenous ventricular morphology Echocardiographic diastolic function and EDP indices exhibit limited values in Fontan patients
	CMR	CMR allows a better quantification of volumes, systolic function and flows
	Cardiac catheterization *(Fontan pathway pressures, PVR, TPG, PAWP, CO)*	Cardiac catheterization should be complemented with fluid challenge or exercise as needed to provide a better characterization of EDP and pulmonary vasculature
**Exercise performance**	CPET 6MWT *(complementary or alternative in patients not capable of performing CPET)*	Progressive decline in exercise capacity yields a more significant prognostic impact than absolute values
**Electrophysiology evaluation**	ICD implantation for sudden cardiac death prevention Cardiac pacing for SND or conduction disorders Suitability for electrophysiological ablation of arrhythmic substrate	Fontan conversion is a high-risk procedure which may be considered in selected patients with refractory arrhythmias
**Residual anatomic lesions (i.e., Fontan pathway obstruction, pulmonary branch stenosis)**	CT Cardiac catheterization	Treatment of residual anatomic lesions should be pursued both for bridging and as an alternative option for AHFT May relevantly affect VAD filling
**Fenestration status**	TEE CMR Cardiac catheterization	Fenestration status is particularly important in VAD candidates CMR allows precise quantification of fenestration flow Creating/reopening in case of elevated CVP-related complications; closing a fenestration in cyanotic patients may be a bridge or an alternative strategy to AHFT according to specific cases
**Lymphatic complications**	Hypogammaglobulinemia, lymphopenia, coagulation, and calcium and bone density assessment in PLE Lymphatic imaging (T2-weighted MRI, lymphangiography)	Lymphatic imaging is particularly important in addressing suitability for percutaneous lymphatic interventions
**FALD**	Hepatic laboratory assessment	Hepatic synthetic deficit has prognostic value, but existing scores present limited value in staging the degree of liver dysfunction for combined liver–heart transplant
	Cross-sectional liver imaging (CT, MR)	Cross-sectional liver imaging is a useful tool for non-invasive diagnosis and staging of liver fibrosis and hepatocellular carcinoma surveillance
	Liver biopsy	Liver biopsy is generally indicated in patients referred for AHFT for fibrosis staging
	Screening for portal hypertension complications (i.e., *varices*, *splenomegaly*)	Hepatic vein pressure gradient is not reliable for diagnosis of portal hypertension in Fontan patients
**CKD**	Serum biomarkers (creatinine, cystatin C, azotaemia) Albuminuria and proteinuria Renal ultrasound with Doppler	Cystatin C correlates more accurately than creatinine with the level of kidney dysfunction to abnormal body composition in Fontan patients
**Pulmonary complications**	PFT CT	Identification and preoperative optimization of pulmonary parenchymal and restrictive disease may help in management of ventilatory support
**Alloantibody sensitization**	HLA antibodies	In PLE, allosensitization may be masked by concomitant hypogammaglobulinemia and lymphopenia, thus necessitating more strict surveillance post-HT
**Nutritional assessment**		Particularly relevant in patients with PLE
**Frailty assessment**		Sarcopenia, disability, comorbidity, and impaired physical and cognitive status
**Social and psychological assessment**		Adherence to medical therapy and follow up Higher rates of anxiety/depression in CHD patients

List of abbreviations: AHFT = advanced heart failure therapy; CHD = congenital heart disease; CT = computed tomography; CMR = cardiac magnetic resonance; PET = cardiopulmonary exercise testing; FALD = Fontan-associated liver disease; HT = heart transplant; HLA = human leukocyte antigen; ICD = implanted cardiac defibrillator; PFT = pulmonary function test; PLE = protein-losing enteropathy; TEE = transoesophageal echocardiography; TTE = transthoracic echocardiography; VAD = ventricular assistance device; 6MWT = 6 min walking test.

**Table 3:** Most commonly adopted ventricular assist devices for long-term mechanical circulatory support.

	Berlin Heart Excor	Heartmate 3	Total Artificial Heart
***Flow***	PF	CF	PF
***Position***	Paracorporeal	Intracorporeal	Intracorporeal
***Configuration***	SVAD, SpVAD, BiVAD	SVAD	BiVAD
***Patient size***	Wide weight range ≥ 3 kg	Approved for BSA ≥ 1.2 m^2^ or weight > 30 kg	TAH 70 cc: BSA ≥ 1.7 m^2^
	*(the most adopted in the pediatric population, especially < 20 kg)*	Off-label use in smaller patients (smallest BSA 0.78 m^2^; weight 19 kg) with higher risk of complications	TAH 50 cc: BSA ≥ 1.2–1.8 m^2^
***Specific issues***	▪ Does not allow discharge in most countries; ▪ Limits mobilization and rehabilitation; ▪ Dedicated Fontan venous cannulas.	Compared to the previously available HVAD (used up to 0.4 m^2^ or 10 kg): ▪ Better haemodynamic profile with higher EDP reduction and lower rate of thrombosis and stroke in standard adults; ▪ Shorter inflow cannula (may reduce suction events); ▪ Larger extracardiac pump (may limit implantation in smaller chests).	▪ High-risk surgery requiring resection of the ventricular mass with various techniques (i.e., *ventricular cuff or total ventricular resection with atrial cuff*); ▪ Limited by available space in the chest (CT mandatory for suitability assessment).

List of abbreviations: BiVAD: biventricular assist device; BSA: body surface area; CF: continuous flow; CT: computed tomography; EDP: end-diastolic pressure; HVAD: Heartware ventricular assist device; PF: pulsatile flow; SpVAD: subpulmonary ventricular assist device; SVDA: systemic ventricle assist device; TAH: total artificial heart.

## Data Availability

No new data were created or analyzed in this study. Data sharing is not applicable to this article.

## Abbreviations

The following abbreviations are used in the manuscript:

AHFT	advanced heart failure therapy
APC	aortopulmonary collateral
AVVR	atrioventricular valve regurgitation
BiVAD	biventricular assist device
BTT	bridge to transplant
BTC	bridge to candidacy
CBP	cardiopulmonary bypass
CC	cardiac catheterization
CHLT	combined heart and liver transplant
CMR	cardiovascular magnetic resonance
CO	cardiac output
CVP	central venous pressure
EDP	end-diastolic pressure
FALD	Fontan-associated liver disease
HF	heart failure
HLA	human leukocyte antigen
HT	heart transplant
MCS	mechanical circulatory support
PAVM	pulmonary arteriovenous malformation
PB	plastic bronchitis
PLE	protein losing enteropathy
PVR	pulmonary vascular resistance
SVAD	systemic ventricular assist device
SpVAD	subpulmonary assist device
VA-ECMO	veno-arterial extracorporeal membrane oxygenation
VAD	ventricular assist device
VVC	veno-venous collateral
